# Cytosolic redox components regulate protein homeostasis via additional localisation in the mitochondrial intermembrane space

**DOI:** 10.1002/1873-3468.12766

**Published:** 2017-08-06

**Authors:** Mauricio Cardenas‐Rodriguez, Kostas Tokatlidis

**Affiliations:** ^1^ Institute of Molecular, Cell and Systems Biology College of Medical, Veterinary and Life Sciences University of Glasgow UK

**Keywords:** mitochondria, oxidative folding, reductive pathways

## Abstract

Oxidative protein folding is confined to the bacterial periplasm, endoplasmic reticulum and the mitochondrial intermembrane space. Maintaining a redox balance requires the presence of reductive pathways. The major thiol‐reducing pathways engage the thioredoxin and the glutaredoxin systems which are involved in removal of oxidants, protein proofreading and folding. Alterations in redox balance likely affect the flux of these redox pathways and are related to ageing and diseases such as neurodegenerative disorders and cancer. Here, we first review the well‐studied oxidative and reductive processes in the bacterial periplasm and the endoplasmic reticulum, and then discuss the less understood process in the mitochondrial intermembrane space, highlighting its importance for the proper function of the cell.

## Abbreviations

**DDP**, deafness dystonia protein

**ER**, endoplasmic reticulum

**FAD**, flavin‐adenine‐dinucleotide

**Glr**, glutathione reductase

**Grx**, glutaredoxins

**IMS**, intermembrane space

**MMP**, matrix mitochondrial peptidase

**NADPH**, nicotinamide‐adenine‐dinucleotide phosphate

**PDI**, protein disulphide isomerase

**ROS**, reactive oxygen species

**TIM**, translocator of the inner membrane

**TNF**, tumour necrosis factor

**Trx**, thioredoxin

The reductive and oxidative (redox) environment of the cell is determined by all the oxidative and reductive reactions that take place in the different cell compartments. Among those reactions, the balance between the production and removal of both reactive oxygen species (ROS) and reactive nitrogen species (RNS) is critical 1, 2. ROS and RNS are products of normal cell metabolism. At elevated levels, they lead to the formation of highly reactive damaging species, while at normal levels, they are involved in beneficial cell signalling and metabolism 3, 4, 5. The maintenance of this balance is important because it directly influences the environment in which critical cellular processes take place, such as the activation of transcription factors, protein proofreading and protein biogenesis 6, 7, 8, 9. The latter is of particular interest for mitochondria, as 99% of all mitochondrial proteins are synthesised in the cytosol. Thus, these proteins need to be imported into mitochondria by highly regulated processes that involve several pathways. The main pathways for mitochondrial protein import are the presequence pathway for proteins targeted to the mitochondrial matrix, and the carrier pathway for inner membrane polytopic proteins.

Most of the mitochondrial protein import pathways require external energy either in the form of ATP hydrolysis or the presence of the inner membrane electrochemical potential. By contrast, the oxidative folding pathway for intermembrane space (IMS) proteins operates without an external energy source and is independent of the inner membrane potential. The mechanistic details of the major energy‐requiring import pathways have been reviewed extensively elsewhere 10, 11, 12.

Oxidative protein folding is the process by which a protein acquires native disulphide bonds and its native conformation. This process of structural disulphide bond insertion takes place not only in the mitochondrial IMS but also in the bacterial periplasm and the endoplasmic reticulum (ER). The machineries in charge of this oxidative folding have been extensively studied in the three compartments. As for the reductive processes, which are necessary for maintaining a redox balance, the periplasm and the ER possess reducing mechanisms which serve primarily for disulphide isomerisation 13, 14. Unlike those compartments, there is no reducing pathway described so far for the IMS. However, cytosolic components of the main disulphide reduction systems, i.e. the thioredoxin and the glutaredoxin systems, have recently been shown to localise also into the IMS 15, 16 raising interesting questions about the feasibility and operation of a reductive pathway in the IMS. In this review, we discuss primarily the oxidative and reductive processes in the IMS and compare them to those operating in the bacterial periplasm and the ER. Additionally, we evaluate the importance on redox homeostasis and putative *modus operandi* of these newly identified members of reducing machineries in the IMS.

## Oxidative folding

**Figure 1:**
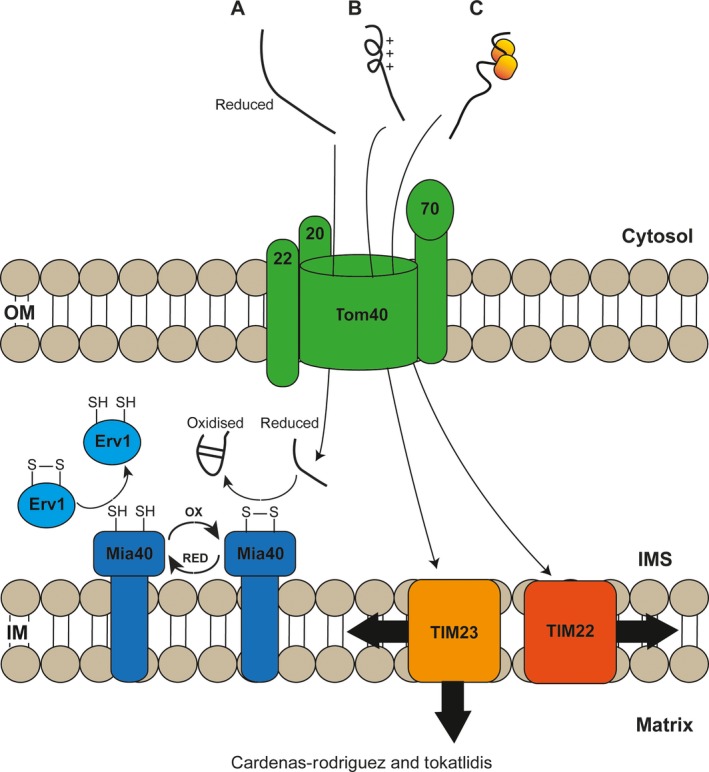
The three main protein import pathways into mitochondria. (A) The MIA pathway precursors cross the OM membrane via the TOM channel in a reduced state. Once in the IMS, they interact with Mia40 which serves as receptor and oxidoreductase and promotes the insertion of disulphide bonds into the proteins. (B) The presequence pathway precursors are translocated across the TOM channel to the TIM23 complex. They contain a positively charged presequence that helps them cross the IM in a membrane potential (ΔΨ)‐dependent manner. These proteins are then either fully translocated to the matrix or transferred to the IM and the presequence is cleaved off. (C) The carrier pathway precursors are first delivered to the Tom70 receptor by cytosolic chaperones. In the IMS, the small Tim proteins help them to interact with the TIM22 complex for their insertion into the IM.

Oxidative protein folding is a process responsible for the insertion of disulphide bonds within proteins that allows them to acquire and/or stabilise their native three‐dimensional structure. It is well established that this process takes place in three different compartments, i.e. the periplasm in bacteria, and the endoplasmic reticulum (ER) and the mitochondrial intermembrane space (IMS) in eukaryotic cells (Fig. 1). In the next section, the oxidative folding pathways in the bacterial periplasm and the ER will be described as a basis to compare them with the IMS pathway.

## Gram‐negative bacteria and ER

Before an oxidative folding pathway was discovered in the mitochondrial IMS, it was believed that oxidative folding occurred only in the bacterial periplasm and the ER of eukaryotic cells. In the Gram‐negative bacterial periplasm, oxidative folding is driven by two proteins: the periplasmic thiol‐disulphide oxidoreductase DsbA (Dsb for di**s**ulphide bond) and the inner membrane protein DsbB. DsbA is a 21 kDa protein member of the thioredoxin family and is one of the most oxidising proteins known so far 17, 18. It contains an active CX_2_C motif placed in a thioredoxin domain that forms a mixed disulphide with the substrate protein which is then released after the formation of an internal disulphide 19, 20. The resulting reduced DsbA is reoxidised by DsbB 21, 22, which transfers e^−^ to quinone and subsequently become oxidised again. Finally, these e^−^ are transferred to oxygen 23, 24. Alternatively, under anaerobic conditions, the e^−^ cascade runs from DsbB onto menaquinone and then to fumarate 23, 24, 25.

The ER is responsible for disulphide formation in a much wider set of proteins than the periplasm or the IMS, including cell surface and secreted proteins such as antibodies. In this compartment, cysteine oxidation is driven by members of the protein disulphide isomerase (PDI) family and by the oxidase Ero1 (ER oxidoreductase 1). PDIs have at least one thioredoxin‐like domain. The main PDI in the ER has four thioredoxin domains, two of which are active CX_2_C domains that can accept e^−^ from the nascent reduced protein 26, 27. Then, the PDI‐reduced cysteines are oxidised by the flavin‐adenine‐dinucleotide (FAD)‐linked sulfhydryl oxidase, Ero1. The catalytic domains of Ero1 internally shuttle the e^−^ onto FAD and this in turn to molecular oxygen, leading to the production of H_2_O_2_ 28, 29, 30, 31, 32.

## The mitochondrial IMS

In mitochondria, import of IMS proteins plays a vital role for the physiology of the cell as they are part of the respiratory chain and are involved in apoptosis, lipid homeostasis and the transport of metabolites, metal ions and haem. There are two main import mechanisms for IMS proteins. One is responsible for the import of proteins containing a mitochondrial targeting presequence followed by a hydrophobic sorting domain. The presequence targets the protein to the translocator of the inner membrane (TIM) 23 complex where it is cleaved off by the matrix mitochondrial peptidase (MMP). The hydrophobic sorting domain functions as a stop‐transfer signal that arrests further translocation into the matrix. A second cleavage just after the stop‐transfer signal takes place in the IMS by the intermembrane space protease IMP that releases the mature protein into this compartment 33. In a separate pathway, IMS proteins that acquire disulphide bonds are imported by the mitochondrial IMS import and assembly (MIA) pathway. The MIA pathway is responsible for the import of most proteins residing in the IMS. The two core components of the MIA pathway are the oxidoreductase Mia40 and the sulfhydryl oxidase Erv1 (essential for respiration and vegetative growth 1) 7, 34, 35. Mia40 is widely conserved among species. It shares with its homologues a characteristic domain about 60 amino acids in length that contains six cysteine residues organised as one CPC (cysteine–proline–cysteine) and two CX_9_C motifs 35, 36. The CX_9_C motifs stabilise the core of the protein and form a hydrophobic cleft that serves to bind and hold substrates, whereas the active CPC motif is the acceptor of e^−^ shuttled from the incoming protein 7, 34, 35, 37. In the same way as the periplasm and ER oxidative pathways, Mia40 is recycled back to an oxidised state by a sulfhydryl oxidase after it oxidises its substrates. Like its ER counterpart, Erv1 (ALR in humans) is a FAD‐coupled sulfhydryl oxidase that accepts e^−^ from Mia40 and transfers them via FAD and cytochrome c to molecular oxygen or an unknown acceptor under aerobic or anaerobic conditions respectively 38, 39, 40. The capacity of the Flavin moiety to assist both two‐ and one‐electron transfer reactions seems to be critical for the operation of the oxidative folding pathways in the different compartments. Although Mia40 and Erv1 are the key proteins (encoded by essential genes) for the oxidative folding pathway in the IMS, another nonessential factor that has an auxiliary role is Hot13. This protein is a cysteine‐rich protein (containing 10 cysteines) and has been proposed to facilitate Mia40 reoxidation by keeping Mia40 in a Zn‐free state thus allowing an easier interaction of Mia40 with Erv1 41, 42.

The classical substrates of the MIA pathway possess either CX_3_C or CX_9_C twin motifs. MIA substrates have an intermembrane space targeting (ITS) signal (also known as mitochondria IMS‐sorting signal, MISS) in addition to the twin cysteine motifs and need to be in a reduced state to cross the translocator of the outer membrane (TOM) channel formed by Tom40 to be imported into mitochondria 43, 44. The proteins of the small TIMs family and members of the COX family are examples of CX_3_C and CX_9_C classical MIA substrates. The small Tims are proteins that form two complexes, the essential Tim10–Tim9 complex and the Tim8–Tim13 complex, to deliver hydrophobic proteins to the TIM22 complex for insertion into the IM 10, 45. Their importance for human cells was corroborated by the finding that mutations of the Tim8 human homologue, the deafness dystonia protein (DDP), are associated with a neurodegenerative disorder, the Mohr‐Tranebjaerg syndrome 46. COX family members are involved with the assembly of cytochrome c, an essential component of the respiratory chain. However, other MIA substrates with different cysteine patterns have also been identified. Examples are the copper chaperone for superoxide dismutase (Ccs) 1 47, the Fe‐S cluster protein Dre2 48 and the inner membrane protease Atp23 49. The identification of these proteins leads to the conclusion that Mia40 has a wider role in protein import, not only as an oxidative molecule but also as a receptor 37, 50. Mia40 itself is not a conventional substrate of Mia40 and in fact its import has been found to occur in three stages. First, it is imported and inserted in the inner membrane by the TIM23 complex, then interaction with the endogenous Mia40 helps the core to fold, and finally the CPC motif is oxidised by an interaction with Erv1 51.

Although the e^−^ cascade is similar in all three oxidative folding systems in bacteria the ER and the IMS, the MIA machinery has different mechanistic properties. Furthermore, the key players Mia40 and Erv1 do not show homology to their periplasm and ER counterparts. This is shown by the short‐term intermediates formed between the substrates and DsbA or PDI as compared to the stable intermediates with Mia40. Another important difference between the three systems relies on the environment where they take place as the IMS is more reducing than the periplasm or the ER. That might explain the reason for more stable intermediates formed by Mia40.

Among those newly identified redox players in the IMS, the thiol peroxidase Gpx3 (also called Hyr1 or Orp1) is of great relevance. Gpx3 is member of the glutathione peroxidase protein family which acts as a sensor for H_2_O_2_. Gpx3 has a critical role in the activation of the H_2_O_2_ response in the cytosol. Upon H_2_O_2_ treatment, Gpx3 becomes oxidised and forms a mixed intermolecular disulphide with the transcription factor Yap1. Subsequently, Yap1 becomes activated through formation of and intramolecular disulphide and transfer to the nucleus. In this mechanism, Gpx3 functions as a sensor and transducer for the hydroperoxide response 52. Recently, it was published that an IMS‐localised Gpx3 form containing an 18‐amino acid N‐terminal extension plays an important role in mitochondria. Cells lacking Gpx3 have defects in morphology and protein import as well as lower inner membrane potential 53.

## Reducing mechanisms

In the three compartments where oxidative folding pathways take place, reducing reactions are key for maintaining the homeostasis between oxidative and reductive flows, and also for the proper functioning of these pathways. In the next paragraphs, we will discuss first the disulphide isomerase machineries that have evolved in the periplasm and the ER, and then the requirement for reducing pathways in the IMS for optimal function of the MIA pathway.

## Isomerisation and reductive pathways in the periplasm and the ER

**Figure 2:**
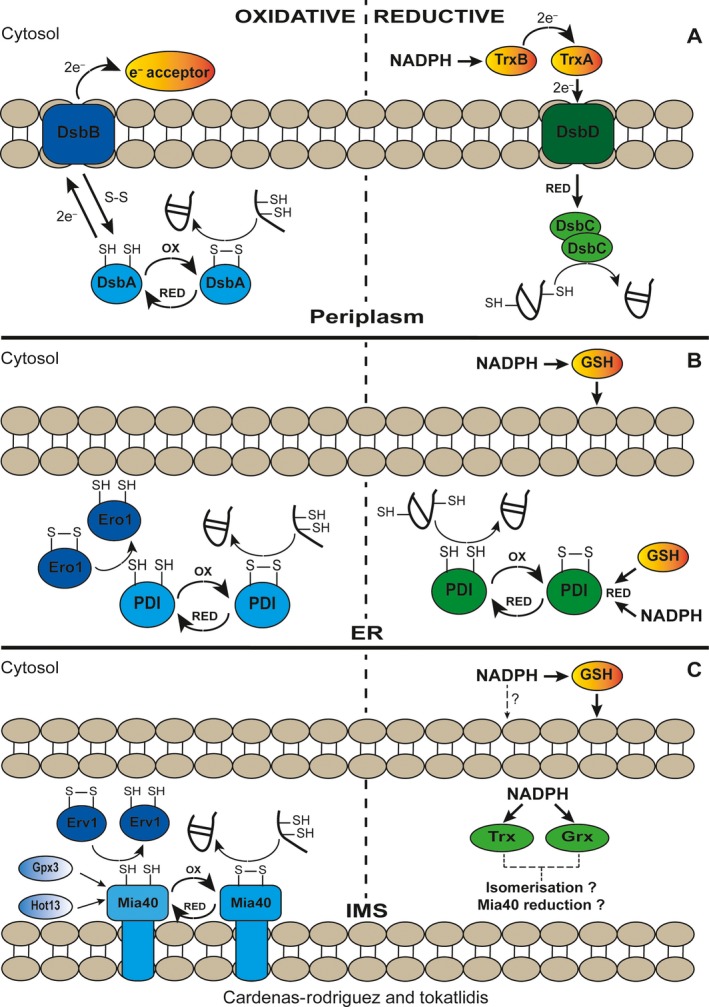
Redox processes in the bacterial periplasm, endoplasmic reticulum and mitochondrial intermembrane space. (A) Redox processes taking place in the bacterial periplasm. Oxidation of proteins by DsbA, which is in turn oxidised by the membrane protein DsbB (left). Electron flux from the cytosolic Trx system onto DsbD, then to DsbC and finally to the protein (right). (B) Redox processes in the ER. Protein oxidation by PDI and reduced PDI recycling by e^−^ transfer onto Ero1 (left). Protein reduction/isomerisation pathway by PDI reduced by either GSH or NADPH (right). (C) Redox processes in the IMS. Protein import mediated by the Mia40 oxidation of protein precursors. Mia40 is kept in an oxidised state by Erv1, or alternatively by Gpx3. This process may be facilitated by Hot13 which is proposed to keep Mia40 in its reduced state ((left). The Trx and Grx systems were recently localised in the IMS. It is likely that these reductive systems play a role in Mia40 reduction to keep the oxidised/reduced overall state of Mia40 necessary for its import function and also that they play a role in protein isomerisation (right).

Thiol oxidation to form disulphide bonds between cysteine residues in a denatured state of a protein is a process prone to error that can be harmful to the cell because of protein misfolding. To prevent this, the bacterial periplasm and the ER possess oxidoreductases that can overcome this problem as they maintain their active sites in a thiol‐reduced state that can cleave non‐native disulphides thereby allowing reformation of the correct ones. Despite differences in the pathways between the two compartments, they both share the reduced form of the cofactor nicotinamide‐adenine‐dinucleotide phosphate (NADPH) as their primary e^−^ donor (Fig. 2).

In the bacterial periplasm, the oxidoreductases DsbC and DsbD are responsible for the isomerisation of non‐native disulphides. DsbC is a 23 kDa homodimer with both isomerase and chaperone activity 54. The isomerisation and thus reduction of aberrant disulphides were shown directly by the accumulation of misfolded proteins in DsbC mutants and the complementation by the addition of the reductive compound dithiolthreitol (DTT) 13. The DsbC thioredoxin domain is kept reduced by the membrane protein DsbD. DsbD is embedded in the inner membrane of *Escherichia coli* with two periplasmic‐exposed domains that transfer e^−^ from NADPH via thioredoxin (Trx) not only to DsbD but also to DsbG as well 55, 56. Even though both the oxidative and reducing mechanisms take place in the same compartment, they are kept kinetically separated to prevent nonfunctional disulphide redox reactions to happen 57.

In the ER, disulphide bond reshuffling is driven by members of the PDI family. The reduction of PDI is mediated by either the interaction with the cysteine residues of newly synthesised polypepetides in the ER lumen or the interaction with reduced glutathione (GSH), which is believed to be transported from the cytosol 31. In the cytosol, GSH is kept reduced by a NADPH‐dependent protein, the glutathione reductase (Glr). This is shown by the fact that the reduction of ERp57, a PDI family member, is prevented by Glr inhibition 58. In contrast to the bacterial periplasm, the oxidative and reductive mechanisms are both performed by PDIs, whose redox state is partially oxidised. The oxidised/reduced ratio of the PDIs dictates its flux towards oxidising or reducing purposes. When this oxidised/reduced ratio is high, disulphide transfer from Ero1 to PDIs is prevented by the formation of intermolecular disulphides within Ero1. When the ratio is low, Ero1 can perform its oxidising function towards PDI 59. Taken together, the periplasm and ER redox machineries, both in their oxidising and reducing state, are key to highly regulated processes that are vital for the cell/organism fate. Furthermore, they have developed different mechanisms to keep them properly working while sharing the same localisation.

The thioredoxin fold is a characteristic motif shared by many proteins involved in thiol‐redox state modification. Those proteins are Dsb proteins, PDI, glutaredoxins and thioredoxins. Its basic structure consists of three α‐helices surrounding four β‐sheets and the active CX_2_C motif. As members of this protein family have different (sometimes opposite) roles, fine modifications (particularly these affecting their redox state) determine their function 60. The different redox properties of these proteins are determined by the type of amino acids between the cysteine couple in the catalytic site, as well as a loop containing a *cis*‐proline 61.

The glutaredoxin system is composed of glutaredoxins (grx), GSH and GSH reductases. Glutathione is a γ‐L‐glutamyl‐L‐cysteinyl‐glycine tripeptide that plays a critical role in redox reactions and is considered as the major nonprotein thiol‐reducing agent. GSH is involved in proteins thiol‐reduction by recycling grx back to a reductive state as well as in redox cell signalling by the modification of cysteines through a process called glutathionylation 62. While GSH keeps grx active (reduced), these proteins are in charge of the reduction of both protein disulphides and protein–GSH mixed disulphides. Grx proteins are a group of widely conserved oxidoreductases of 10–15 kDa with thioredoxin fold‐like domains. They can be classified into two categories based on their active site: monothiol (CX_2_S) and dithiol (CX_2_C). Together with their e^−^ donor role, they are also involved in the regulation of cellular oxidative stress response. These proteins are involved in several cellular processes such as transcription, cellular differentiation, apoptosis and Fe‐S cluster coordination, and some human isoforms have been found to be overexpressed in cancer 63, 64, 65, 66, 67. In the same way as the oxidoreductases within the periplasm, the ER and the IMS, grx needs to be recycled back to its active reduced state after intramolecular disulphide formation. As mentioned before, GSH is responsible for the reduction of grx disulphide. The resulting glutathione oxidised form (GSSG) is then reduced by the NADPH‐fuelled protein, glutathione reductase.

**Figure 3:**
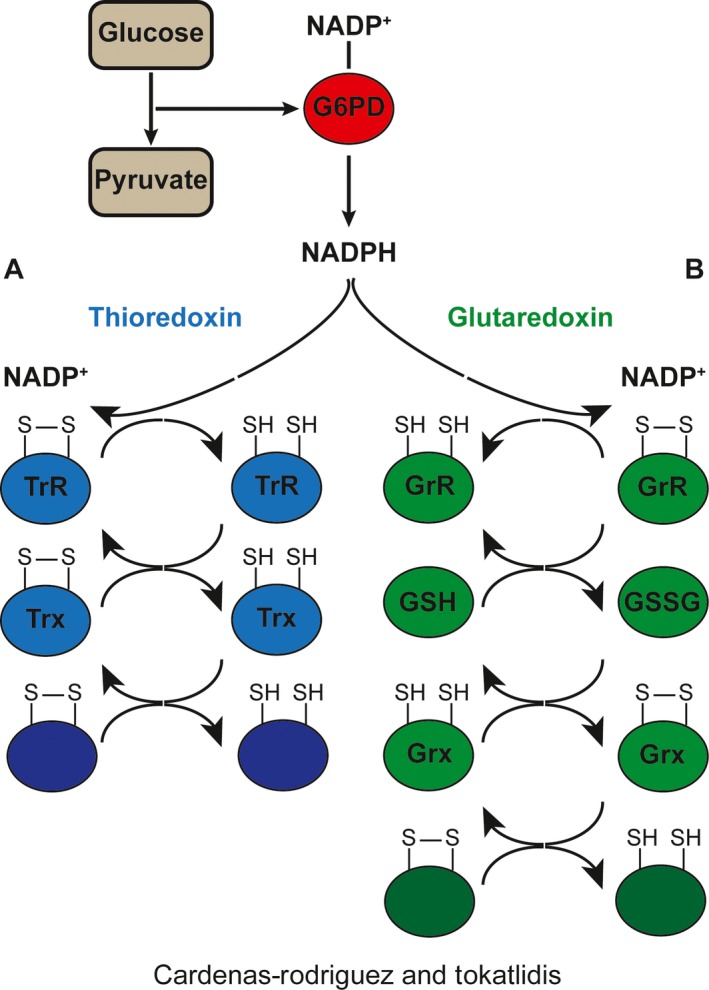
The two main thiol‐reductive systems. Both the thioredoxin and the glutaredoxin system use the NADPH produced by the pentose phosphate pathway enzyme G6PD as their final electron donor. (A) The thioredoxin system electron flux starts from NADPH which reduces the thioredoxin reductase, which then reduces the thioredoxin protein. Finally, the thioredoxin protein transfers this e^−^ to its substrates. (B) The glutaredoxin system follows a similar e^−^ flux with an extra step involving glutathione reductase e^−^ transfer onto glutathione, and subsequently, from glutathione onto glutaredoxin and then to glutaredoxin substrates.

The thioredoxin system is present in all organisms and comprises the highly conserved trx protein, trx reductase (trxR) and NADPH. The trx system works in a similar way as the grx system, where trx transfers e^−^ onto the substrate through thiol‐disulphide exchange reactions and is critical for maintaining the redox balance of the cell. Those e^−^ follows a cascade from NADPH to trxR and subsequently to trx. This system is involved in a wide range of pathways. It is the donor of reducing equivalents for the peptide methionine sulfoxide reductase, whose reductase activity upon methionine reverses the inactivation of several proteins 68. Additionally, trx has shown inhibitory activity on the tumour necrosis factor (TNF) α‐induced activation of the apoptosis signal‐regulating kinase (ASK) 1, which has been related to several diseases such as cancer and neurodegenerative disorders 69. Other critical processes such as cell growth, transcription, apoptosis and DNA synthesis among other functions nonrelated to its redox capacity have been reported 70, 71. Trx is the defining member of the thioredoxin protein family. It was first identified as e^−^ donor for ribonucleotide reductase in *E. coli* 72. Alike grx, its reducing activity relies on the cysteine pair of its catalytic CX_2_C domain. TrxR is a flavoprotein that catalyses the reduction of trx. There are two forms of TrxR, a 35 kDa protein in prokaryotes and a 50 kDa in higher eukaryotes 73, which are structurally different with the active site located in the FAD‐binding domain in the eukaryotic form or in the NADPH‐binding domain in the prokaryotic one 74, 75 (Fig. 3).

## The IMS: shared cytosolic‐reducing pathways

Unlike the bacterial periplasm and the ER, there are no reducing pathways described so far for the IMS. Nevertheless, the importance of the presence of reducing systems is overriding for the redox homeostasis to be kept so it is plausible that the IMS possess such reductive systems. In fact, proteins from the two main thiol‐reducing families, the thioredoxin and glutaredoxin systems that are mostly localised in the cytosol, were recently found to be present in the IMS of the yeast *Saccharomyces cerevisiae* using high‐resolution mass spectrometry and biochemical analysis 15, 16. As shown in the other cellular subcompartments where oxidative folding takes place, the redox homeostasis has a critical role as the oxidative pathways need the complementation of reducing pathways in order to work properly and keep the cell well‐being. The influence on the reducing pathways into mitochondrial IMS starts with the fact that protein precursors need to be in a reduced state in order to cross the OM. This latter was shown by the facilitated import of small Tims precursors into mitochondria after treatment with purified Trx system 76. Although this external influence is important, the compartmentalised redox balance of the IMS is the actual key player. Although there is no reducing mechanism in the IMS described so far, the recent identification of several redox‐related proteins with dual cytosolic and IMS localisation has been reported in several studies. Mechanisms for dual localisation of some mitochondrial proteins have been reviewed elsewhere 77. Proteins belonging to the thioredoxin family and the peroxiredoxin family might have redox roles in the IMS 78.

Furthermore, other studies have shown that the GSH redox potential of the IMS and the cytosol are linked and that this has an impact on the redox state of Mia40 79. Additionally, the presence of glutaredoxins within the IMS was demonstrated raising the possibility that these proteins may be linked to the oxidative folding process in the IMS. This could be achieved by ensuring the presence of an appropriate mixed oxidised/reduced Mia40 population balance, which is necessary for the proper function of Mia40 16. Of particular importance is also the reported presence of Trx1 and TrR1 in the IMS proteome. Although the exact function of this system in the IMS is currently unknown, the matrix‐located thioredoxin system (Trx3 and Trr2 in *S. cerevisiae*) was shown to modulate the redox state of the mitochondrial matrix protein cyclophilin D, a protein involved in the regulation of the mitochondrial permeability transition pore 80.

Despite the identification of reducing small molecules and proteins within the IMS, and the hints given for their putative mechanistic and regulatory role in the interplay with oxidative folding, no clear pathway has been yet described in this cellular subcompartment. Given the intriguing localisation of these components in the IMS and the similarities between the IMS and both the bacterial periplasm and the ER, it is likely that complete reducing machineries operate in the IMS compartment. These reducing machineries might play important roles within the mitochondrial IMS to keep the redox balance necessary for the proper function of the oxidative import pathways. The relative GSH equilibrium between the cytosol and the IMS and the Mia40 redox state regulation by Grx give some initial insight on the roles these systems can perform as the Trx and Grx systems can compensate each other when one of them is defective. Another key issue to clarify will be the link of the IMS reducing systems to the overall cellular metabolic state, given that the main NADPH source resides outside the IMS (in the cytosol) in the form of the glucose‐6‐phosphate dehydrogenase (G6PD), the rate limiting enzyme of the pentose phosphate pathway. Indeed, unpublished data from our group show import defects in mitochondria from yeast cells lacking G6PD. The identification and characterisation of the Trx and Grx pathways as well as the impact of cytosolic redox and metabolic changes on them will be important to fully comprehend the links among the mechanism of oxidative folding, protein homeostasis and redox signalling in the mitochondria intermembrane space.
